# Embodied Metarepresentations

**DOI:** 10.3389/fnbot.2022.836799

**Published:** 2022-04-28

**Authors:** Nicolás Hinrichs, Maryam Foradi, Tariq Yousef, Elisa Hartmann, Susanne Triesch, Jan Kaßel, Johannes Pein

**Affiliations:** ^1^Faculty of Philology, Institute for Applied Linguistics and Translatology, Leipzig University, Leipzig, Germany; ^2^Natural Language Processing Group, Department of Computer Science, University of Leipzig, Leipzig, Germany; ^3^Chair of Software Engineering, Faculty of Computer Science, Chemnitz University of Technology, Chemnitz, Germany; ^4^Department of Scandinavian and Finnish Studies, University of Cologne, Cologne, Germany; ^5^Department of German Linguistics, German Studies, Heinrich Heine University Düsseldorf, Düsseldorf, Germany; ^6^Institute of Indology and Central Asian Studies, Universität Leipzig, Leipzig, Germany; ^7^Faculty of Mathematics and Computer Science, Leipzig University, Leipzig, Germany

**Keywords:** translation universals, natural semantic metalanguage (NSM), natural language processing (NLP), semantic mirroring, FrameNet, frame semantics, 4EA cognition, semantic frame parsing

## Abstract

Meaning has been established pervasively as a central concept throughout disciplines that were involved in cognitive revolution. Its metaphoric usage comes to be, first and foremost, through the interpreter's constraint: representational relationships and contents are considered to be in the “eye” or mind of the observer and shared properties among observers themselves are knowable through interlinguistic phenomena, such as translation. Despite the instability of meaning in relation to its underdetermination by reference, it can be a *tertium comparationis* or “third comparator” for extended human cognition if gauged through invariants that exist in transfer processes such as translation, as all languages and cultures are rooted in pan-human experience and, thus, share and express species-specific ontology. Meaning, seen as a cognitive competence, does not stop outside of the body but extends, depends, and partners with other agents and the environment. A novel approach for exploring the transfer properties of some constituent items of the original natural semantic metalanguage in English, that is, semantic primitives, is presented: FrameNet's semantic frames, evoked by the primes SEE and FEEL, were extracted from EuroParl, a parallel corpus that allows for the automatic word alignment of items with their synonyms. Large Ontology Multilingual Extraction was used. Afterward, following the Semantic Mirrors Method, a procedure that consists back-translating into source language, a translatological examination of translated and original versions of items was performed. A fully automated pipeline was designed and tested, with the purpose of exploring associated frame shifts and, thus, beginning a research agenda on their alleged universality as linguistic features of translation, which will be complemented with and contrasted against further massive feedback through a citizen science approach, as well as cognitive and neurophysiological examinations. Additionally, an embodied account of frame semantics is proposed.

## Introduction

### Carrying Across

Meaning has been pervasively established as a central concept throughout disciplines that were involved in cognitive revolution (Pylyshyn, 1984). Its metaphoric usage comes to be, first and foremost, through the interpreter's constraint: representational relationships and contents are considered to be in the “eye” or mind of the observer, and shared properties between observers themselves are knowable through interlinguistic phenomena, such as translation (Semin and Smith, 2007). Despite the instability of meaning in relation to its underdetermination by reference (Baumgarten, 2012)^1^, it can be a *tertium comparationis* for extended human cognition (Hatim and Munday, 2019)^2^ if gauged through invariants that exist in transfer processes such as translation, as all languages and cultures are rooted in pan-human experience and, thus, share and express species-specific ontology.

Translation and interpreting studies (TISs) regard translation as a carrying-across task or an act of transfer with the aim of “communicating the overall meaning of a stretch of language” (Baker, 2018: 10). The notion of meaning is central to this definition, and it stems directly from it being considered as the primordial invariant in lexical semantics, allowing for its consideration as “third comparator.” Meaning, seen as cognitive competence (Barsalou, 1992), does not stop outside of the body but extends, depends, and partners with other agents and the environment (Rojas-Líbano and Parada, 2020). Indeed, according to embodied theories of cognition (4EC), an epistemological view that has had considerable acceptance in TISs, human cognition and, by extension, the act of translation and interpreting are considered to be embedded, extended, embodied, and enacted (Muñoz Martín, 2017).

### Embodied Metarepresentations

The collaboration among psychology, linguistics, neuroscience, computer science, anthropology, and philosophy yielded a new metalanguage (Araneda Hinrichs, 2020), wherein these scientific enterprises immersed to the point of not noticing its metaphorical nature. To this day, artificial intelligence, digital humanities, and even neurorobotics still rely on the symbolic-computational paradigm, which was born under the umbrella of information processing theory.

The assumption of this metaphor is that representation is constituted as some form of encoding, that is, as physicalist mapping of correspondences between mental states of an agent and actual things in the world. By intrinsically restricting research to issues of manipulation and transformation of already constituted carriers of representational content, meaning was replaced with data; thus, the fundamental problem of representation ceased to be addressed (Brette, 2019), that is, the interactive emergence and function of representational content (Meteyard et al., 2012).

Labeling and the subsequent exploratory modeling of embodied metarepresentations for representational correspondences that are necessary, irreducible and, moreover, shared through culture and sociality (Goddard, 2010), understood as dynamic couplings of agents (Enfield and Kockelman, 2017), might aid in breaking out of the symbolic-computational paradigm and serve as a theoretical middle ground for computational and cognitive linguistics in order to leverage the notion of them being shared cultural artifacts that will allow for the exploration of the emergence of multilingual semantic relations.

A novel approach for exploring the transfer properties of semantic frames (SFs) is presented, on the basis of some constituent items of the original natural semantic metalanguage (NSM) in English (Wierzbicka, 1996), that is, semantic primes or primitives (SPs). Focusing on the SPs SEE and FEEL, SFs that their verb instances evoked in actual language use were identified in the European Parliament Proceedings Parallel Corpus (EuroParl). This parallel corpus (Leech, 1991; Kay and Röscheisen, 1993; Véronis, 2000) allows for the automatic word alignment of items with their translations (Koehn, 2005), and SF annotation was conducted using the state-of-the-art FrameNet parser (Xia et al., 2021), a tool for multilingual frame-semantic annotation to semi-automatically query the search of prototypical patterns of translational text production (Alves and Vale, 2017). Afterward, semantic mirroring (SM) (Vandevoorde, 2020), a procedure that consists back-translating into source language, allowed for the translatological examination of translated and original versions of the items.

Frameshifts associated with SM were observed and described using a fully automated pipeline, which was designed and tested for the purpose of facilitating the exploration on the question of the alleged universality of certain frameshifts as linguistic features of translation. This approach will be complemented with and contrasted against a citizen science approach to annotation correction by means of massive online feedback. The claim that said shifts are tended toward ubiquity in a human-translated language will be challenged from an enactive perspective and empirically examined through cognitive and neurophysiological experiments.

### Theoretical Background

#### Priming Primitives

The NSM is based on the hypothesis that languages have a universal core in common. There's a semantic struggle to define the concrete meanings of words without being trapped in circular descriptions. As Wierzbicka (1996) points out, “to *demand* is defined as 'to request firmly', and to *request* as 'to demand gently;”' to solve such a problem, it seems that we need to break through the circular structures of definitions. Referring to Wierzbicka, therefore, we need to set elements that “can be used to define the meaning of words (or any other meaning)” on the one hand. On the other hand, they should not be able to define themselves. These elements are to be marked as “indefinibilia,” and these, in turn, are summarized as a set of primitives.

Moreover, Wierzbicka (1996: 11) argues that “any set of primitives is better than none, because without some such set semantic description is inherently circular and, ultimately, untenable. This does not mean, however, that it is a matter of indifference what set of primitives one is operating with, as long as one has some such set. Far from it: the best semantic descriptions are worth only as much as the set of primitives on which they are based. For this reason, for a semanticist the pursuit of an optimal set of primitives must be a matter of first importance.”

The suggested list of primitives by Wierzbicka consists of 63 semantic primes, of which 13 are verbs. Frame semantics and its application in FrameNet focus on verbs as frame-evoking elements (Atkins et al., 2003, 252), drawing on their rich syntactic and semantic valency. For this study, two verbs out of the NSM were chosen, namely, SEE and FEEL, for the reason that these are associated with the psychophysical act of seeing through one's eyes, as well as them being able to be used in order to express sensations or opinions and to verbalize the realization of events.

Goddard and Wierzbicka (2014) point out common complications that may come forth when identifying exponents of primes, such as polysemy (i.e., two primes that share a single exponent) and allolexy (i.e., a given prime that has different exponents in different contexts), while there are numerous examples of further ones, such as portmanteau exponents (i.e., combinations of primes that can be expressed by means of a single word). This study focused on two specific primitives and their respective particular exponents: the form of the verbs SEE and FEEL, as they were uttered in EuroParl, in order to probe the proposed pipeline, granted that the polysemous use of both of them thwarts their association as primes to specific frames. Indeed, psychophysical acts linked to primitive SEE are only recognizable cross-linguistically through the perception_experience frame.

#### Framing Frames

Frame semantics (FS) demands the entanglement of sociality and meaning. According to Barsalou's (1992) frame theory, for instance, all representations of objects and categories in human cognition are exclusive in terms of functional concepts (i.e., attributes of frames assign values to their arguments or, in other words, frames are recursive attribute-value structures). In FS, several different theories and approaches can be identified, with Barsalou's theory of concept frames differing from Fillmore's (1968) approach, which focuses more strongly on linguistic instantiations of frames, especially on predicates (cf. Busse, 2012). For this study, the latter theory and its application in the FrameNet project will be central, as it lends itself to a semi-automatic study on language data.

FS explains the complex system of relations that a speaker must know in order to understand an utterance. In a nutshell, understanding of a given meaning emerges from a broader understanding of a state of affairs, building on world knowledge, and prior experience, a word can be said to represent a category of experience (Petruck, 1997: 1). Semantic frames are schematic representations of situations, “story fragments” (Ruppenhofer et al., 2016: 7), mental systems of concepts that structure world knowledge and experience, thus facilitating understanding.

For example, we can understand the predicate “buy” in *She bought a new bike* only if we are familiar with the system of concepts at work in a commercial transaction, that there is a buyer and a seller who exchange goods for money. This world knowledge and its linguistic instantiations are modeled in the frame Commercial_transaction with four core frame elements (FEs), buyer, seller, goods, and money. Activating any one of these concepts makes all the others readily available in the minds of speakers and hearers (Petruck, 1997: 1).

Frames are inherently cross-cultural and cross-linguistically applicable, albeit differences exist especially with culturally bound concepts, e.g., in societal domains such as law, with consequences for frame semantic databases in different languages (Bertoldi and Chishman, 2012). In translation, semantic frames are at the core of the meaning that is carried across. Czulo (2017) proposes a *primacy of frame model* building on “the idea that preserving the conceptual information connected with a frame in the source language by picking an adequate frame in the target language is a core procedure in translation” (Czulo, 2013: 144). While picking an *adequate* frame will, in many cases, mean choosing the *maximally comparable frame* to the one in the source text, a number of factors can override this principle and bring about frameshifts (Czulo, 2017: 479).

SFs are, by definition, based on conceptual structures (Fillmore, 1968), which constitute generalizations over surface structure and, therefore, ought to be less prone to syntactic variation (Padó and Lapata, 2009). Boas (2005) has proposed FS as an interlingual meaning representation, and research suggests the plausibility of recurrent usage of SFs originally modeled for English in other languages (Ohara et al., 2003; Subirats and Petruck, 2003; Burchardt et al., 2009).

Manual annotation and lexicographic work based on the FrameNet approach (more details below) have been applied to many other languages, with FrameNets existing, for example, for German, Brazilian, Portuguese, and Swedish. The Global FrameNet initiative was launched to organize and bring all existing FrameNets under one umbrella aiming at development of collaborative research and shared tasks (Global FrameNet, 2021). However, the field of automatic frame annotation in a multilingual context is still not well-practiced. This is under way, and with the novel FrameNet parser, a tool for automatic, multilingual frame-semantic annotation, perspectives for the development of such a tool are getting more realizable (Xia et al., 2021).

The interplay of translational divergences (van Leuven-Zwart, 1989; Dorr, 1995) and frame dis-/agreements has begun to be examined (Padó and Lapata, 2009), and measures such as Frame Match (FM), for cases where same SFs are evoked in different languages, have been defined.

#### Semantic Mirroring

Semantic mirroring (SM) was devised originally by Dyvik (1998, 2005) as an automated method for deriving large-scale entries from a word-aligned parallel corpus for machine translation and other kinds of multilingual processing; Vandevoorde (2020) extended this technique for the analysis of sets of lexemes as representations of semantic fields, thus enabling their comparison, as it allows one to statistically visualize semantic relations in translated and untranslated language as well as their distances. The presented pipeline takes inspiration in SM as a means to consider the variation of frames evoked by original, translated, and back-translated sentences as clusters of meaning distinctions, so as to raise questions pertaining to the nature and characteristics of the observed tendencies.

Variation is inherent in translation, it is the rule rather than the exception to have a variety of more or less equally valid possibilities of translating a given utterance, and it is all about choices being made by the translator who takes into consideration a number of factors from the overall skopos of the translation (Reiß and Vermeer, 1984) to micro-level lexicalization.

In machine translation (MT), it is no longer a human being making these decisions but statistical models or artificial neural networks trained on data produced by human translators. While MT is more prone to errors or inappropriate translation solutions for the lack of “real” (i.e., human) understanding and creativity, there are systems available today that provide high-quality translations for certain language pairs and text domains. The notion and measuring of translation quality are important issues in TIS, with models being developed both for human translation (e.g., House, 1997) and machine translation (e.g., the BLEU score for automatic evaluation of MT output by comparing it to professional human translations; Papineni et al., 2002). The data used here comprises both professional human translations (EuroParl) and machine-translated data (back-translation using DeepL).

## Materials and Methods

### Corpus

As mentioned above, EuroParl V7^3^ was employed for this study; more specifically, the corpora in English, German, and Spanish. EuroParl is a parallel corpus with translations provided by professional human translators and is extracted from the European Parliament website by Koehn (2005). This corpus was chosen in light of its “(…) free availability, size, linguistic diversity, data authenticity, and sentence-aligned architecture as well as homogeneity in terms of register, text type, and subject domain (…)” (Ustaszewski, 2019: 107), all of which make it ideal for translation-oriented corpus-based inquiries, moreover, if applied as a data-driven approach that serves to characterize mental or sociocultural aspects of interlingual phenomena.

Europarl consists of two pieces: the unprocessed source obtained from the European parliament and the processed and aligned sentence-by-sentence output for each language pair. The raw source material contains additional metadata *via* XML-like pieces of meta-information. Utterances of each speaker are structured in paragraphs, with one text file containing a day's worth of paragraphs. The meta-information covers information on chapters (i.e., their ID), paragraphs, and speakers (i.e., their ID, name, and language). The entire corpus was post-processed by iterating over the source in order to acquire these pieces of information and attach them to each utterance of the aligned corpus. Using this corpus required some pre-processing and several decision-making procedures for filtering and querying relevant sentences, which will be discussed and explained further below.

### Instruments

#### Word Alignment

Translation alignment is the process of comparing two texts in different languages and finding translation equivalences between the tokens in the source text and their correspondences in the translation. It is essential in neural and statistical machine translation, cross-lingual annotation projection (David et al., 2001; Padó and Lapata, 2009), and translation lexica induction (Yousef, 2020). Previous models have employed unsupervised statistical methods to generate alignment probability distribution between the source and target textual units (Brown et al., 1993; Och and Ney, 2003; Dyer et al., 2013).

With the advent of deep learning models and transformer language models, pre-trained word embeddings are being used to capture translation correspondences among tokens in the source text and its translation (Sabet et al., 2020; Dou and Neubig, 2021). In this article, word alignment was captured by computing the similarity of contextualized multilingual word embeddings among the tokens to the parallel sentences using the multilingual Bert language model (Devlin et al., 2018) fine-tuned to the present parallel data set, in a similar vein to Dou and Neubig (2021), who reported an increase in the quality of alignment output compared to previous models.

#### DeepL Translator

DeepL^4^ is a state-of-the-art neural machine translation system that provides an automatic translation service launched in 2017. With its high translation quality, especially for European languages, DeepL performed all other competing tools. The quality of DeepL translations has been studied very frequently through different evaluation approaches (e.g., manually using Human Translation Edit Rate (HTER) or automatically with the likes of BLUE score, for instance) confirming the outperformance of this translation engine compared to other freely accessible ones (Kur, 2019; Bellés-Calvera and Quintana, 2021).

#### Berkeley FrameNet

The online database FrameNet^5^ is a lexical resource applying the theory of frame semantics to contemporary English (Petruck, 1997; Fillmore et al., 2003; Ruppenhofer et al., 2016). The aim of the Berkeley FrameNet (BFN) project is to “document the range of semantic and syntactic combinatorial possibilities, valences, of each word in each of its senses, through computer-assisted annotation of example sentences and automatic tabulation and display of the annotation results” (Ruppenhofer et al., 2016: 7, bold in original). The current seventh release of BFN provides definitions for more than 1,000 frames along with their frame elements and frame evoking elements, adding up to more than 13,600 lexical units (LUs) with an average of 20.8 annotation sets per LU^6^. A lexical unit is defined as a pairing of a word with a meaning, with each sense of a polysemous word typically belonging to a different semantic frame (Ruppenhofer et al., 2016: 7). Frames are interrelated, forming a network, and information on frame-to-frame relations is included in each frame entry.

While BFN is based on English, frames are considered cross-linguistically applicable to a certain extent. Thus, FrameNet frames have been used for developing lexical databases and annotated corpora for different languages, specialized domains, or even interlingual representations for multilingual representations (Boas, 2005). A range of NLP applications has been developed that draw on BFN data, e.g., automatic semantic role labeling (ASRL) and applications in deep semantic analysis of texts (Petruck, 2011).

#### Large Multilingual Information Extraction

LOME is an end-to-end multilingual frame semantic parsing model (Xia et al., 2021, recently made available and performs all three traditional steps of the frame semantic parsing process, namely, target/predicate identification, frame identification, and semantic role identification (Minnema, 2021). LOME offers a state-of-the-art trained model for neural FrameNet parsing with an accuracy higher than 91%. LOME uses XML-R (Conneau et al., 2019) as its underlying encoder, which enables it to perform very well on multilingual data. Although this tool offers various annotations including semantic frames and lexical units, for this study, the focus was put on parsed semantic frames for the verbs SEE and FEEL.

### Pipeline

**Figure 1:**
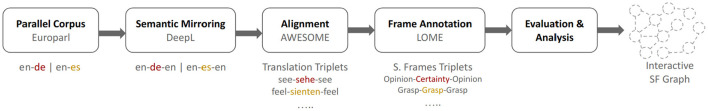
Pipeline of the steps undertaken for frame extraction and comparison.

Corpus-based studies constitute a data-driven approach to characterize mental or sociocultural aspects of interlingual phenomena through massive collections of translated texts. Thanks to digital tools, translation-process data can be queried semi-automatically in search of prototypical patterns of text production (Alves and Vale, 2017). As Leech (1991: 11) puts it, “any kind of specialized language (represented in a domain-specific corpus)” can be chosen to profit from its richness in semantic variation across lexeme translation. Consequently, EuroParl was chosen, although it is somewhat restricted in genre (i.e., transcriptions of spoken text and limited to political context), it is based on professionally translated proceedings of the European Parliament, aligned at both the document and sentence levels as can be seen in Figure 1.

Europarl is subject to a pre-processing procedure that takes place during the alignment that includes stripping of punctuation. Hence, many sentences from the source do not fully match their aligned counterparts (e.g., when long sentences in German have to be translated into several English sentences while interpreting). Instead of re-processing the entire source with the provided software and attaching meta-information, the readily processed output was iterated over line-by-line in search for the respective source sentence within the source corpus. Then, the meta-information was searched for within the proximity of that sentence by iterating in reverse order, since the meta-information will be provided before the utterance. Using eurol1^7^ enabled us to process the Europarl parallel corpus in the previously described way, which enriches the corpus with meta-information.

This meta-information is vital for this study given that EuroParl is a database that consists of sentences that were either uttered in an L1 or translated from any of the 24 official languages of the European Union. The meta-information was used to filter out the ones for which it was not specified whether their L1 was English. Unfortunately, this information on the Europarl parallel corpus covers only parts of the sentences. For the DE-EN aligned corpus, about 50% of the sentences do not have an original language attached with them (980,917 of 1,920,209 sentences). Of these, only 2,277 are stated to be uttered in German, 68,291 in Spanish, and 232,878 in English.

After filtering the corpus for sentences with English as L1, a dataset of English sentences containing one of the conjugated forms of SPs SEE and FEEL was collected. These sentences were aligned with their German and Spanish translations using the word alignment model described above. In this way, it was observed how each token was translated into German and Spanish. Because of the authentic nature of the translations performed by interpreters of the European Parliament, it is evident that not all the instances of both verbs have a one-to-one correspondence; thus, the data had to be further sorted by filtering out these sentences, for which there was no clear correspondence in the target language; for instance, a frequent case consisted of a verb being aligned with a NULL value or a preposition. Table 1 illustrates the final quantities of the sentences that were used for the analysis.

**Table 1:** Frequency of the sentences used in the final analysis.

	**# Sentences**		**Verb**	**# Sentences**	**# Sentences**
EN-DE	4,528	See	See	2,362	3,577
			Seen	859	
			Sees	94	
			Saw	262	
		Feel	Felt	212	951
			Feel	658	
			Feels	81	
EN-ES	3,503	See	See	1,872	2,912
			Seen	801	
			Saw	156	
			Sees	83	
		Feel	Felt	149	591
			Feel	371	
			Feels	71	

Having ~4,500 sentences for the language pair EN-DE and 3,500 sentences for EN-ES, DeepL was used for their back-translation into English. In order to find translations of the corresponding verbs in the back translation, however, another step of word alignment was conducted. In this way, the data set of sentences in English was prepared as source language, translations into German or Spanish and back-translations of these sentences into English for semantic parsing through LOME. As mentioned before, LOME annotates a whole sentence with corresponding frames evoked in that sentence and related lexical units.

**Figure 2:**
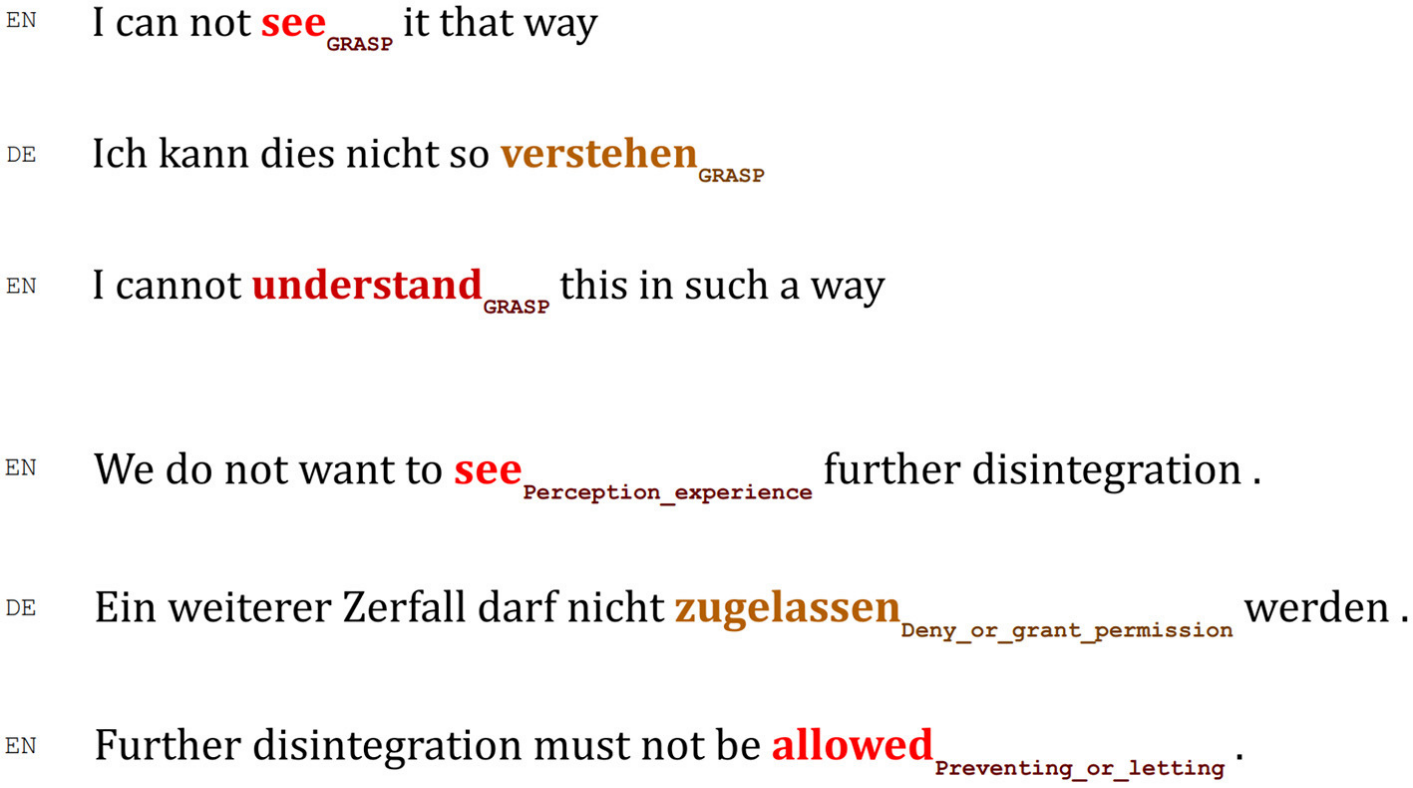
Word aligned and semantic frame tagged sentences for language pair English-German.

Since in this study the focus was put on two verbs, word alignment enabled the extraction of only frames that were evoked by each verb, namely, SEE and FEEL. This allowed for the visualization of their variation across original English, translations into German and Spanish, and their back-translations into English. Figure 2 illustrates the results of word alignment and semantic frame annotation processes described in this section for the language pair English-German. As this Figure indicates, the frames can stay consistent, or a frameshift after each translation iteration can be observed.

## Results and Discussion

Because of the explorative nature of this research, in this section, the aim lies in describing the findings with the support of some visualizations. These visualizations allow for the observation of frameshifts and, accordingly, the shift in meaning, so as to make a basic comparison between verbs and languages. Additionally, a statistical analysis is presented, as it further helps in understanding and evaluating these findings.

At the outset, LOME labeled the verb FEEL with 98 frames for the language pair English-German and 53 frames for the language pair English-Spanish. The number of detected frames for the verb SEE was 222 frames in English-German and 194 frames in English-Spanish datasets. One of the reasons for this difference in the number of detected frames roots back to the number of analyzed sentences (see Table 1). It is to be noted, as Table 2 indicates, that frame combinations as well as frameshifts with high frequencies were rather scarce; thus, for practical purposes, only these were selected to be included in the visualizations.

**Table 2:** Most frequently detected and labeled frames in each data set.

	**Frame**	**Source**	**%**	**Translation**	**%**	**Back translation**	**%**
See (EN-DE)	Perception_experience	1,966	58.69%	930	32.75%	993	37.26%
	Grasp	1,025	30.60%	257	9.05%	524	19.66%
	Categorization	182	5.43%	351	12.36%	199	7.47%
	Becoming_aware	87	2.60%	383	13.49%	156	5.85%
	Causation	42	1.25%	13	0.46%	18	0.68%
	Others	248	1.43%	906	31.90%	775	29.08%
Feel (EN-DE)	Opinion	587	62.25%	181	27.38%	272	43.66%
	Feeling	292	30.97%	99	14.98%	114	18.30%
	Perception_experience	39	4.14%	37	5.60%	18	2.89%
	Give_impression	9	0.95%	3	0.45%	3	0.48%
	Experiencer_focus	3	0.32%	4	0.61%	11	1.77%
	Others	13	1.38%	337	50.98%	205	32.91%
See (EN-ES)	Perception_experience	1,744	64.78%	960	39.69%	1,245	52.29%
	Grasp	666	24.74%	164	6.78%	380	15.96%
	Categorization	134	4.98%	222	9.18%	155	6.51%
	Becoming_aware	50	1.86%	310	12.82%	81	3.40%
	Causation	47	1.75%	10	0.41%	29	1.22%
	Others	51	1.89%	753	31.12%	491	20.62%
Feel (EN-ES)	Opinion	287	48.89%	109	21.25%	119	26.44%
	Feeling	243	41.40%	167	32.55%	140	31.11%
	Perception_experience	42	7.16%	28	5.46%	33	7.33%
	Give_impression	7	1.19%	31	6.04%	22	4.89%
	Grasp	1	0.17%	2	0.39%	3	0.67%
	Others	7	1.19%	176	34.31%	133	29.56%

### Visualizations

**Figure 3:**
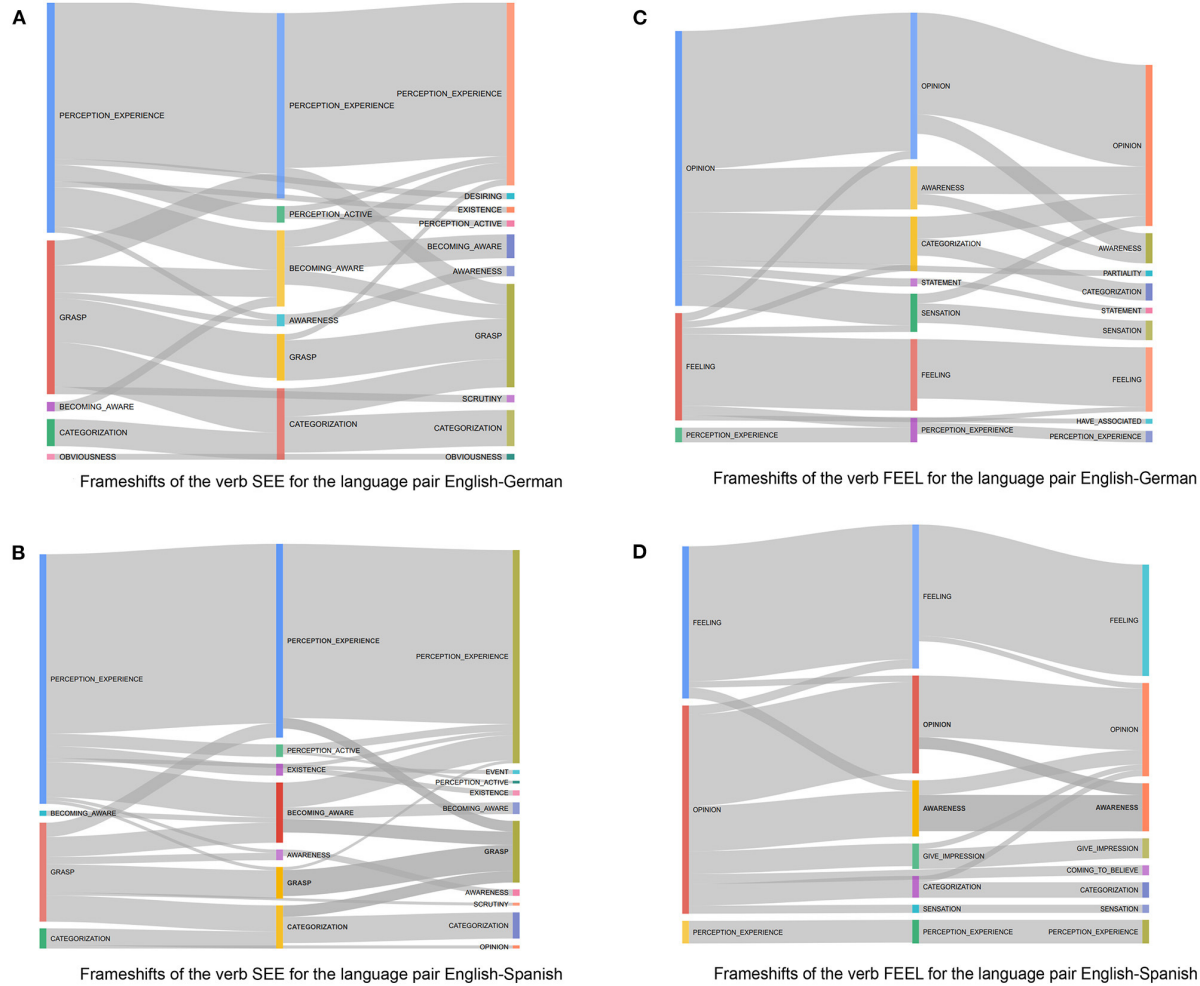
**(A)** Frameshifts of the verb SEE for the language pair English-German. **(B)** Frameshifts of the verb SEE for the language pair English-Spanish. **(C)** Frameshifts of the verb FEEL for the language pair English-German. **(D)** Frameshifts of the verb FEEL for the language pair English-Spanish.

As mentioned in the previous sections, the following visualizations enable us to observe frameshift patterns for both verbs FEEL and SEE while being translated from English into German and Spanish and back-translated from these two languages into English. Figure 3A gives an overview of the most frequent patterns for the verb SEE in the English-German data set, while Figure 3B represents the patterns of frameshifts of this verb in the English-Spanish data set. Tables 4, 5 presented in Appendix also summarize the frequencies of frame combinations and frameshifts when the verb SEE is being translated into German and Spanish and being back-translated into English.

For SEE, the single most often evoked frame is perception_experience in all the three language versions both for the German and the Spanish subcorpora; however, after being translated, not all the evoked frames are limited to perception_experience^8^. For example, out of 1,996 occurrences of perception_experience in the source text for language pair German-English (Table 3), only in 722 cases a consistent pattern can be observed (Table A4), where the frame does not change in the path of translation and back-translation.

**Table 3:** Manual evaluation of large ontology multilingual extraction (LOME)^9^.

**Language**	**No frame**	**Correct**	**Possible**	**Incorrect**	**#Sentences**	**Accuracy1**	**Accuracy2**
English	27	137	22	14	200	91%	92%
German	8	29	7	6	50	82%	85%
Spanish	8	30	10	2	50	94%	95%
All	43	196	39	22	300	90%	91%

The same pattern is also observable in the visualization 3b referring to the verb SEE in the English-Spanish data set. However, in the English-Spanish dataset, a frameshift to grasp can be detected in few cases, while this frameshift does not occur in the English-German data set. perception_active, becoming_aware, and awareness are frames that have been commonly evoked in the translated sentences into both German and Spanish.

The most frequent frame in the source text is followed by grasp. This is little surprising, considering first that SEE denotes visual perception and, second, its metaphorical use as the concept of understanding. Interestingly enough, it can be observed again that in both languages the frame grasp tends to be shifted into other frames, and it is less consistent than the frame perception_experience. This can be clarified by the definition of this frame:

- Grasp: a cognizer possesses knowledge of the working, significance, or meaning of an idea or object, which is referred to as phenomenon, and is able to make predictions about the behavior or occurrence of the phenomenon. The phenomenon may be incorporated into a wider knowledge structure *via* categorization, which can be indicated by the mention of a category.

Moreover, a case could be made for the frame Grasp being evoked as the conceptual metaphor of Understanding is seeing as one that holds an embodied grounds to this meaning.

Even though no frame-to-relation for grasp and categorization is defined in FrameNet, categorization can be considered a more specific instance of grasp. Grasp includes the possibility of a phenomenon being categorized, and that could be the reason for the frameshift observed in the data sets.

The other interesting phenomena that can be detected with the help of presented visualizations is that even though the tendency of a frameshift from perception_experience into becoming_aware is seen during its back-translation into German and Spanish, in comparison to the other frameshifts, the probability of a reverse frameshift from becoming_aware into perception_experience in the back translation is relatively high.

Because of the literal nature of machine translations, frameshifts would not be expected in this case. What stands out and can be seen in the visualizations is that this is not necessarily the case. Moreover, the frame becoming_aware is related to the discovery of and/or finding out about something (according to FrameNet, words in this frame are related to a cognizer adding some phenomenon to their model of the world). This raises the question whether or to what extent the lexical aspect of language or the translators' cognition related to it could be affecting the frameshifts.

As indicated in Figures 3C,D, the two most prominent frames for FEEL in all language combinations are feeling and opinion. Interestingly, their proportion appears to be flipped, comparing the German and Spanish translations. As with SEE, it is little surprising to see the frame motivating the basic denotation of the verb to be among the most frequently annotated ones. However, two interesting observations can be reported.

First, the most frequent frame in the source English is opinion, a cognizer holds a particular opinion, which may be portrayed as being about a particular topic, which definitely can be explained by the fact that in a political discourse (EuroParl corpus) the verb FEEL is mostly used to express an opinion and not a feeling, which is when an experiencer experiences an emotion or is in an emotional_state. Secondly, although in German the frame Opinion shifts to Awareness, Categorization and Statement, shifts to Sensation.

The fact that the opinion frame features so prominently in the annotation can be explained by the domain of the corpus data: expressing opinions can be expected as a key element in political speeches. Other senses of “feel” evoking other frames listed in FrameNet relate to physical actions such as the active perception of touching something or searching for something by feeling for it. These frames are to be expected in rather different text types, e.g., narrative instead of political speeches. A manual analysis showed that the word *Gefühl* has been labeled by the frame sensation, which, in this case, refers to an error of a fully-automated parser.

In summary, in all the four analyses, the frames associated most closely with the denotation of the target expression (perception_experience for SEE and feeling for FEEL) show few frameshifts. The frame motivating another sense of the verb undergoes considerably more frameshifts.

These observations allow us to formulate a hypothesis, which needs to be analyzed in further steps of this research, and that is whether or not the lexical resources for expressing this second sense differ more strongly cross-linguistically. The questions of whether or not there are different aspects and factors that lead to more variations in the translation, and how these can be explained by a cognitive analysis of the translation process evidently become a fundamental interest.

### Evaluation of the Automatic Tools

While the present pipeline incorporated state-of-the-art resources such as automatic translation (DeepL), translation alignment [i.e., AWESOME, developed by Dou and Neubig (2021)], and the SF parser (LOME) (Xia et al., 2021), these tools are automated neural models that will never reach human performance. To get a realistic picture of their performance, they were manually evaluated.

As indicated in Table 3, to assess the performance of the alignment model, the alignment of over 100 sentences selected randomly from the data set was manually evaluated. For the evaluation, each annotation was labeled as correct, partially correct, or incorrect. The evaluation showed that the accuracy of the alignment reached 92% for English-German-English sentences and 97% for English-Spanish-English.

Additionally, the performance of LOME on over 100 parallel sentences (source, translation, and back translation) was manually evaluated, resulting in 300 sentences (200 English sentences, 50 German sentences, and 50 Spanish sentences). LOME performed on the Spanish sentences with 95% accuracy, 92% on the English sentences, and 85% on the German sentences; the overall performance on over 300 sentences was 91%.

The sample provided examples for a variety of problems that the automatic tools had. One problem is the lack of frame semantic annotation for the targets in the translated and/or back-translated sentences. The possible reasons lie in the way the automatic tools are interrelated in the pipeline: while LOME, in principle, provides annotations for whole sentences, target expressions were selected following those which the alignment tool identified as translations of the primitives SEE and FEEL. In a number of cases, semantically weak or empty elements such as auxiliary verbs (1), particles, or even punctuation signs in the translation or back-translation were aligned to the respective source expression. Obviously, such elements are no viable candidates for frame-semantic annotation. In other cases, the alignment worked well, but LOME did not provide annotation for the target expression.

The manual evaluation suggests that LOME accuracy rates are about as high for the original English sentences as stated as general accuracy in Xia et al. (2021). Looking at the annotation of the translated and back-translated sentences, the accuracy slightly decreases, especially when taking into account the proportion of zero annotations, but becomes higher when more steps of translation are performed to the sentences.

### Detailed Analysis of Frame Combinations

The aim of this section is to provide some examples to explain the reasons why frame detection was incorrect in some cases and why no frames for some specific verbs were detected. Additionally, with the help of additional examples from the data sets, it is showcased how frames remain consistent or to what extent a frameshift can be observed. These examples allow us to have a better understanding of the above presented visualizations.

In (1), the phrase “we saw huge tranches of her own report being deleted” was translated into German leaving out the aspect of “seeing,” thus presenting the event of deletion without the additional layer of perception. Consequently, the automatic tools could neither detect a direct translation of “saw” to be aligned to the source expression nor provide a frame semantic annotation.

(1)  
EN_S_Only 2 weeks ago in Parliament, we saw_Perception_experience_ huge tranches of her own report being deleted, because they contained references to such a body.
DEErst vor zwei Wochen wurden große Teile ihres Berichtes hier im Parlament gestrichen, weil sie Bezug auf eine solche Funktion nahmen.
EN_BT_ Only a fortnight ago, large parts of their report were deleted here in Parliament, because they referred to such a function.

Translating instances of the verb FEEL with multi-word units featuring a noun that is the frame-evoking element may or may not cause problems for the alignment and annotation tools: in (2), “feel” was successfully aligned with “*Ansicht*” and the latter with “believe,” and all the three were correctly annotated with the same frame (opinion).

In (3), however, the noun “*Auffassung*” that evokes the same frame did not get aligned to the English target and did not receive frame-semantic annotation. Instead, the verb “*vertritt*” was aligned with the verb in the English source and the back-translation.

(2) 
EN_S_ We are strongly against the trafficking of all people and slavery, but we do not feel_OPINION_ that it is the competence of the EU to interfere in domestic issues, and in particular we do not feel that the EU should be creating a policy regarding prostitution.
DE Wir lehnen den Handel mit allen Menschen und die Sklaverei strikt ab, sind jedoch nicht der Ansicht_OPINION_, dass die EU berechtigt ist, sich in einzelstaatliche Probleme einzumischen. Vor allem sind wir nicht der Ansicht, dass die EU eine Politik im Hinblick auf die Prostitution erarbeiten sollte.
EN_BT_ We are strongly opposed to the trafficking of all human beings and to slavery, but we do not believe_OPINION_ that the EU has the right to interfere in national problems. In particular, we do not believe that the EU should draw up a policy on prostitution.

(3) 
EN_S_ But our group feels_OPINION_ there is tremendous potential.
DE Unsere Fraktion vertritt jedoch die Auffassung, daß dort ein großes Potential liegt.
EN_BT_ However, our group believes_Awareness_ that there is great potential there.

For the vast majority of instances of SEE in the English-Spanish subcorpus examined here, no frameshifts were identified, and all the three language versions are annotated evoking the semantic frame perception_experience. Sentence (4) is an example of this stable relation, featuring high values for semantic similarity (0.9934) and lexical overlap (0.77).

(4) 
EN_S_ I know this is a difficult dossier, which all of us would like to see_Perception_experience_ resolved some way or another.
ES Sé que este es un expediente difícil que a todos nosotros nos gustaría ver_Perception_experience_ resuelto de un modo u otro.
EN_BT_ I know this is a difficult dossier that all of us would like to see_Perception_experience_ resolved in one way or another.

In other cases, the automatic frame semantic annotation is incomplete and/or incorrect because of alignment difficulties. Similar to example (1), the aspect of perception is not expressed, neither in the Spanish translation nor in the English back-translation.

(5) 
EN_S_(5) We do not want to see_Perception_experience_ further additions without a quite specific procedure subject to codecision once more before they are authorized.
ES No queremos que vengan a sumarse nuevos productos sin que se siga un procedimiento específico que esté sujeto a la codecisión, una vez más, antes de ser admitidos.
EN_BT_ We do not want new products to be *added*_Distributed_position_^10^ without following a specific procedure, which is subject to co-decision, once again, before being admitted.

## Conclusions and Outlook

Cross-lingual meaning shifts, albeit a complex and heterogeneous set of phenomena, can be fruitfully studied through the semantic frame theory, as implemented by FrameNet. The presented pipeline probed the stability of semantic primitives by quantitative analysis of human and machine translations, proving frame semantics a useful framework to make an attempt at observing variants and invariants of meaning through semi-automation. Verb instances of SEE and FEEL, elements of NSM, were identified in the EuroParl corpus (which allows for cross-lingual alignment) and annotated for the semantic frames they evoke with use of the LOME tool.

Several frameshifts were observed from a few major frames such as opinion toward a variety of other frames in the translations (both in German as in Spanish), which will require a dedicated qualitative analysis in order to be accounted for.

On the basis of some of the observations detailed in the preceding section, a number of hypotheses can be made, which are relevant to the field of TIS:

1. In the case of the frameshift opinion to awareness, it could be argued that the translator possibly chose a more general expression that less clearly points to a proposition or content being an opinion but constructing it as something that the cognizer/speaker knows/thinks.
2. Regarding the same case, it could also be put forward, following FrameNet, that there is a significant similarity to these frames: “That frame opinion indicates that the cognizer considers something as true, but the opinion (compare to content) is not presupposed to be true; rather it is something that is considered a potential point of difference, as in the following:
  *I think that you are awesome*. In the uses that will remain in the awareness frame, however, the content is presupposed.”
  Thus, the two frames differ in whether or not the content is presupposed. It is possible that the employed tool for automatic annotation did not always capture this somewhat subtle and context-sensitive difference.
3. Considering the shift from the frame opinion to categorization, similarly to the case of grasp to categorization as evoked by the verb SEE, this could possibly be interpreted as a kind of specification or explicitation; that is, instead of “I feel this is provocative,” perhaps “*Ichbetrachte das alsprovokant*” is a case from general FEEL as categorization to a more specific verb expressing categorization.

The initial statistical analyses were limited to the most frequent frame combinations or frameshifts. The opposite cases, which were discarded as outliers, will have to be examined as well, although the annotation quality of the pipeline will need to be revised prior to this once more.

An interactive visualizer is being developed that can be used for display of frames, as well as sentences with which these frames are evoked. It will be further improved through a pipeline that considers behavioral and neurophysiological research and will incorporate a user-friendly interface, so as to enable private research projects and clear cross-cultural understanding of a sentences' underlying meaning.

The exploratory nature of the approach taken in this study was decidedly so in order to observe the usefulness of a fully automated pipeline that would enable an in-depth analysis of frameshifts. The workflow described allowed for its successful testing on the basis of just two SPs that puts forward the potential of this tool, and subsequent analyses will be needed for the entirety of SNM so as to explore it as a suitable basis for its theoretical consideration in relation to upcoming neurocognitive experiments.

So-called universal tendencies of translated texts will be examined as part of a series of upcoming revision iterations of these datasets. Baker (1996) argued for their universality and these includes the “shining through” of the source language (e.g., the near-synonymous convergence of non-synonymous lexemes and vice versa), or its virtual opposite (Hansen-Schirra, 2011) known as normalization or conservatism (i.e., to exaggerate features of the target language, thus conforming to its typical patterns), explicitation and simplification, which mostly stand in relation to lexical variety and/or density, and leveling out -that is, the gravitation of these kinds of text toward the center of a continuum in terms of the Gravitational Pull Hypothesis (GPH) (Halverson, 2003, 2010), according to which salient linguistic items are more likely to be chosen by speakers and, thus, become over- and under-represented.

While the specific typology available in rich corpora such as Europarl was used, widely different mental and sociocultural processes, as well as cognitive operations, involved in its production obscured the interpretations of these results. The findings will, thus, need to be further validated. Problems with the quality of automatic alignment and frame semantic annotation that were identified by manually evaluating a data sample could be avoided by relying less on automatic tools. A small-scale study including human back-translation (by independent translators), manual correction of the alignment, and manual frame semantic annotation would be informative for an overall comparison of results especially for cases that are problematic in the automatic setting (e.g., not showing any frame semantic annotation). Also, word embeddings will be performed in the next study, in order to identify annotations across all the three languages, and a high-dimensional vector space for visualization (i.e., semantic spaces) of their relative relatedness will be developed to facilitate their cross-linguistic comparison.

In relation to the addressed limitation of this study's ecological validity, a digital platform that enables crowdsourcing feedback will be implemented in order to incorporate massive human correction that involves speakers of diverse linguistic and cultural profiles. Using such a method would be an asset, as it allows for larger amounts of linguistic data to be contrasted with manual evaluation.

Additional investigations on the observed frameshifts in the present data should also take frame relations into account. Computing distances between frames based on the frame relation data in BFN would yield information on potential patterns of frameshifts. Approaches and applications of measuring frame distances that could be used for this endeavor are laid out in Minnema and Nissim (2021) and Czulo et al. (2019).

Upon successful testing of the present pipeline against a series of further examinations, which will be designed from an embodied and enactive approach, the definition of frame that incorporates the notion of sociality's entanglement with meaning will have been effectively broadened. This perspective will build on the account of Barsalou (1992), for whom frames are recursive structures that map the functionality of representations; these correspond with culturally constructed affordances, as they are engaged and implemented during phenomena of sociality.

This implies the idea that at the center of cognition lies the concept of intersubjective engagement, where interaction is regarded as mutually regulated dynamic couplings, based on the recognition of the autonomy of interaction that stems from the precariousness of self-regulation. Further analyses of additional corpora and a number of psycholinguistic tasks will be designed for subsequent neurocognitive assessments on the produced material in order to explore factors that might lead to variation in translation processes such as cognitive resources involved in the cross-linguistic task known as “carrying across.”

## Data Availability Statement

The raw data supporting the conclusions of this article will be made available by the authors, without undue reservation.

## Author Contributions

NH and MF conceptualized the present work. NH, MF, EH, TY, and ST co-wrote both the original and current and reviewed version of the manuscript for its publication. NH, MF, TY, and ST performed formal analysis. JK and JP provided initial data curation. All authors contributed to the article and approved the submitted version.

## Funding

The author(s) acknowledge support from the German Research Foundation (DFG) and Universität Leipzig within the program of Open Access Publishing or Dieser Artikel wurde durch den Publikationsfonds der Universität Leipzig und das Programm Open Access Publizieren der DFG gefördert.

## Conflict of Interest

The authors declare that the research was conducted in the absence of any commercial or financial relationships that could be construed as a potential conflict of interest.

## Publisher's Note

All claims expressed in this article are solely those of the authors and do not necessarily represent those of their affiliated organizations, or those of the publisher, the editors and the reviewers. Any product that may be evaluated in this article, or claim that may be made by its manufacturer, is not guaranteed or endorsed by the publisher.

## Footnotes

^1^In a sense to similar Quine's notion of impossibility of radical translation, that is, since reference always underdetermines meaning, one can never be sure of what another says.

^2^As Hatim and Munday (2019: 31) explain, the *tertium comparationis* is based on the idea “that an invariant meaning exists that can be used to gauge or assist transfer of meaning”.

^3^ http://www.statmt.org/europarl/

^4^ www.deepl.com

^5^Freely accessible at framenet.icsi.berkeley.edu.

^6^Status updates at https://framenet.icsi.berkeley.edu/fndrupal/current_status.

^7^ github.com/jankaszel/eurol1

^8^Definitions and annotation data for each frame can be found in FrameNet: https://framenet.icsi.berkeley.edu/fndrupal/frameIndex.

^9^Accuracy1 ignores the examples where the frames are possibly correct, whereas accuracy 2 considers the possibly correct frames as correct.

^10^The automatic annotation for the target “added” in the back-translation is incorrect, instead the frame Cause_to_be_included is evoked.
