# Supplementary material for: Embodied Metarepresentations
Source: Front Neurorobot. 2022 Apr 28;16:836799. doi: 10.3389/fnbot.2022.836799 (PMC9097574; doi:10.3389/fnbot.2022.836799)
Supplement: Supplementary file 1 [file Presentation_1.pdf]

Appendix

Table 4: The frequency of triple frame combination and frameshifts with each translation iteration for verb SEE in English-German dataset.

S-T-B	Freq	S-T	Freq	T-B	Freq
Perception_experience__Perception_experience__Perception_experience	663	Perception_experience__Perception_experience	748	Perception_experience__Perception_experience	722
Grasp__Grasp__Grasp	172	Grasp__Grasp	205	Grasp__Grasp	188
Grasp__Categorization__Grasp	116	Grasp__Categorization	207	Categorization__Grasp	132
Categorization__Categorization__Categorization	100	Categorization__Categorization	126	Categorization__Categorization	168
Grasp__Perception_experience__Grasp	66	Grasp__Perception_experience	116	Perception_experience__Grasp	96
Perception_experience__Becoming_aware__Perception_experience	60	Perception_experience__Becoming_aware	184	Becoming_aware__Perception_experience	77
Grasp__Categorization__Categorization	59	Grasp__Categorization	207	Categorization__Categorization	168
Grasp__Becoming_aware__Grasp	53	Grasp__Becoming_aware	127	Becoming_aware__Grasp	66
Perception_experience__Becoming_aware__Becoming_aware	51	Perception_experience__Becoming_aware	184	Becoming_aware__Becoming_aware	111
Grasp__Perception_experience__Perception_experience	35	Grasp__Perception_experience	116	Perception_experience__Perception_experience	722
Grasp__Becoming_aware__Becoming_aware	31	Grasp__Becoming_aware	127	Becoming_aware__Becoming_aware	111
Perception_experience__Perception_active__Perception_experience	25	Perception_experience__Perception_active	77	Perception_active__Perception_experience	30
Becoming_aware__Becoming_aware__Becoming_aware	25	Becoming_aware__Becoming_aware	44	Becoming_aware__Becoming_aware	111
Perception_experience__Awareness__Awareness	23	Perception_experience__Awareness	28	Awareness__Awareness	48
Grasp__Awareness__Awareness	22	Grasp__Awareness	28	Awareness__Awareness	48
Perception_experience__Perception_active__Perception_active	22	Perception_experience__Perception_active	77	Perception_active__Perception_active	29
Perception_experience__Perception_experience__Grasp	21	Perception_experience__Perception_experience	748	Perception_experience__Grasp	96

Table 5: The frequency of triple frame combination and frameshifts with each translation iteration for verb FEEL in English-German dataset.

S-T-B	Freq	S-T	Freq	T-B	Freq
Opinion__Opinion__Opinion	123	Opinion__Opinion	170	Opinion__Opinion	125
Feeling__Feeling__Feeling	66	Feeling__Feeling	88	Feeling__Feeling	73
Opinion__Awareness__Opinion	33	Opinion__Awareness	53	Awareness__Opinion	34
Opinion__Categorization__Opinion	25	Opinion__Categorization	59	Categorization__Opinion	27
Opinion__Opinion__Awareness	21	Opinion__Opinion	170	Opinion__Awareness	24
Opinion__Sensation__Sensation	19	Opinion__Sensation	39	Sensation__Sensation	24
Opinion__Categorization__Categorization	18	Opinion__Categorization	59	Categorization__Categorization	21
Perception_experience__Perception_experience__Perception_experience	12	Perception_experience__Perception_experience	18	Perception_experience__Perception_experience	14
Opinion__Awareness__Awareness	12	Opinion__Awareness	53	Awareness__Awareness	13
Opinion__Sensation__Opinion	11	Opinion__Sensation	39	Sensation__Opinion	12
Feeling__Perception_experience__Feeling	6	Feeling__Perception_experience	12	Perception_experience__Feeling	6

Table 6: The frequency of triple frame combination and frameshifts with each translation iteration for verb SEE in English-Spanish dataset.

S-T-B	Freq	S-T	Freq	T-B	Freq
Perception_experience__Perception_experience__Perception_experience	788	Perception_experience__Perception_experience	858	Perception_experience__Perception_experience	832
Grasp__Grasp__Grasp	114	Grasp__Grasp	135	Grasp__Grasp	125
Perception_experience__Becoming_aware__Perception_experience	97	Perception_experience__Becoming_aware	167	Becoming_aware__Perception_experience	119
Categorization__Categorization__Categorization	71	Categorization__Categorization	81	Categorization__Categorization	125
Grasp__Becoming_aware__Grasp	51	Grasp__Becoming_aware	96	Becoming_aware__Grasp	64
Grasp__Categorization__Grasp	51	Grasp__Categorization	125	Categorization__Grasp	55
Grasp__Categorization__Categorization	51	Grasp__Categorization	125	Categorization__Categorization	125
Perception_experience__Perception_active__Perception_experience	34	Perception_experience__Perception_active	60	Perception_active__Perception_experience	35
Grasp__Perception_experience__Grasp	33	Grasp__Perception_experience	68	Perception_experience__Grasp	51
Perception_experience__Becoming_aware__Becoming_aware	31	Perception_experience__Becoming_aware	167	Becoming_aware__Becoming_aware	56
Grasp__Perception_experience__Perception_experience	25	Grasp__Perception_experience	68	Perception_experience__Perception_experience	832
Grasp__Awareness__Awareness	23	Grasp__Awareness	35	Awareness__Awareness	31
Grasp__Becoming_aware__Perception_experience	19	Grasp__Becoming_aware	96	Becoming_aware__Perception_experience	119
Perception_experience__Existence__Perception_experience	19	Perception_experience__Existence	57	Existence__Perception_experience	19
Perception_experience__Existence__Existence	16	Perception_experience__Existence	57	Existence__Existence	25
Perception_experience__Perception_experience__Grasp	15	Perception_experience__Perception_experience	858	Perception_experience__Grasp	51

Table 7: The frequency of triple frame combination and frameshifts with each translation iteration for verb FEEL in English-Spanish dataset.

**S-T-B**	**Freq**	**S-T**	**Freq**	**T-B**	**Freq**
Feeling__Feeling__Feeling	113	Feeling__Feeling	149	Feeling__Feeling	123
Opinion__Opinion__Opinion	65	Opinion__Opinion	101	Opinion__Opinion	68
Opinion__Awareness__Awareness	31	Opinion__Awareness	50	Awareness__Awareness	40
Perception_experience__Perception_experience__Perception_experience	25	Perception_experience__Perception_experience	25	Perception_experience__Perception_experience	26
Opinion__Give_impression__Give_impression	18	Opinion__Give_impression	25	Give_impression__Give_impression	22
Opinion__Awareness__Opinion	16	Opinion__Awareness	50	Awareness__Opinion	16
Opinion__Categorization__Categorization	13	Opinion__Categorization	24	Categorization__Categorization	17
Opinion__Opinion__Awareness	13	Opinion__Opinion	101	Opinion__Awareness	13
Feeling__Awareness__Awareness	8	Feeling__Awareness	12	Awareness__Awareness	40
Opinion__Sensation__Sensation	8	Opinion__Sensation	9	Sensation__Sensation	9
Opinion__Categorization__Opinion	7	Opinion__Categorization	24	Categorization__Opinion	7
Opinion__Give_impression__Opinion	6	Opinion__Give_impression	25	Give_impression__Opinion	6
Opinion__Feeling__Feeling	6	Opinion__Feeling	10	Feeling__Feeling	123
